# “Heidelberg standard examination” and “Heidelberg standard procedures” – Development of faculty-wide standards for physical examination techniques and clinical procedures in undergraduate medical education

**DOI:** 10.3205/zma001053

**Published:** 2016-08-15

**Authors:** C. Nikendei, P. Ganschow, J. B. Groener, S. Huwendiek, A. Köchel, N. Köhl-Hackert, R. Pjontek, J. Rodrian, F. Scheibe, A.-K. Stadler, T. Steiner, J. Stiepak, J. Tabatabai, A. Utz, M. Kadmon

**Affiliations:** 1 Heidelberg University Hospital, University Medical Center, Internal Medicine II, Department of General Internal Medicine and Psychosomatics, Heidelberg, Germany; 2 Heidelberg University Hospital, Department of General, Visceral and Transplantation Surgery, Heidelberg, Germany; 3 University Hospital of Munich, Department of General, Visceral, Transplantation, Vascular and Thoracic Surgery, Munich, Germany; 4 Heidelberg University Hospital, University Medical Center, Internal Medicine I, Department of Endocrinology, Metabolism and Clinical Chemistry, Heidelberg, Germany; 5 University of Bern, Institute of Medical Education, Department of Assessment and Evaluation, Bern, Switzerland; 6 Heidelberg University Hospital, Center for Child and Adolescent Medicine, Clinic 1, Heidelberg, Germany; 7 Heidelberg University Hospital, Department of General Medicine and Health Services Research, Heidelberg, Germany; 8 University Hospital RWTH Aachen, Department of Diagnostic and Interventional Neuroradiology, Department of Neurology, Aachen, Germany; 9 Ortenau Hospital Offenburg-Gengenbach, Department of Cardiology, Pneumology, Angiology and Intensive Care Medicine, Offfenburg-Gengenbach, Germany; 10 Klinikum Frankfurt Höchst, Department of Neurology, Frankfurt/Main, Germany; 11 Heidelberg University Hospital, University Medical Center, Internal Medicine III, Department of Cardiology, Angiology and Pneumology, Heidelberg, Germany; 12 Heidelberg University Hospital, Department of Pediatrics I, Center for Child and Adolescent Medicine, Heidelberg, Germany; 13 Ortenau Hospital Offenburg-Gengenbach, Department of Gynecology, Offenburg-Gengenbach, Germany; 14 Carl von Ossietzky University of Oldenburg, School of Medicine and Health Sciences, Oldenburg, Germany

**Keywords:** undergraduate medical education, physical examination, clinical procedures, faculty development

## Abstract

The competent physical examination of patients and the safe and professional implementation of clinical procedures constitute essential components of medical practice in nearly all areas of medicine. The central objective of the projects “Heidelberg standard examination” and “Heidelberg standard procedures”, which were initiated by students, was to establish uniform interdisciplinary standards for physical examination and clinical procedures, and to distribute them in coordination with all clinical disciplines at the Heidelberg University Hospital. The presented project report illuminates the background of the initiative and its methodological implementation. Moreover, it describes the multimedia documentation in the form of pocketbooks and a multimedia internet-based platform, as well as the integration into the curriculum. The project presentation aims to provide orientation and action guidelines to facilitate similar processes in other faculties.

## Zusammenfassung

Die kompetente körperliche Untersuchung von Patientinnen und Patienten und die sichere und professionelle Durchführung klinischer Prozeduren stellen in nahezu allen medizinischen Fachbereichen einen essentiellen Bestandteil des ärztlichen Handelns dar. Die zentrale Zielsetzung der durch Studierende initiierten Projekte „Heidelberger Standarduntersuchung“ und „Heidelberger Standardprozeduren“ war die Etablierung eines einheitlichen interdisziplinären Standards zur körperlichen Untersuchung und zu klinischen Prozeduren und deren Distribution in Abstimmung mit allen klinischen Disziplinen an der Universitätsklinik Heidelberg. Der vorgestellte Projektbericht beleuchtet den Hintergrund der Initiative, die methodische Umsetzung und stellt die multimediale Dokumentation in Form von Kitteltaschenbüchern und einer multimedialen internetbasierten Plattform sowie die curriculäre Integration vor. Die Projektvorstellung soll eine Orientierung und Handlungsleitlinie bieten, um ähnliche Prozesse an anderen Fakultäten zu erleichtern.

## Introduction

The competent physical examination of patients constitutes an essential component of medical practice in nearly all areas of medicine [1]. Together with history-taking, it leads to crucial action-guiding hints for a targeted diagnosis and treatment of the patient [2], [3]. Upon entering the profession, every physician therefore needs to have mastered physical examination to a high level [4]. The situation is similar with respect to the implementation of clinical procedures such as drawing blood, IV cannulation, or mask ventilation. Here, the thorough preparation of materials, knowledge about the order of relevant sub-steps, focused work under sterile conditions, and the correct implementation of the clinical-technical skill are crucial factors for the success of the treatment measure and for patient safety [5]. To achieve this, students not only have to develop the necessary complex psychomotor skills, but also learn the required communicative and affective competences in their interaction with patients, as well as the concluding assessment of findings.

Investigations in medical students show that considerable deficits exist, both in conducting the physical examination and evaluating findings, and in carrying out clinical procedures [6], [7], [8], [9]. A study among students in the Practical Year (PY) at a German university revealed that in the physical examination of four important organ systems, only 40% of the students (thyroid 38%, heart 37%, lungs 42%, abdomen 43%) correctly implemented the a priori defined relevant sub-steps [10]. Likewise, in the interpretation of cardiac and pulmonary auscultation findings [11], pathological findings were only correctly classified in 20 - 45% of cases, which can be seen as posing a considerable danger for misdiagnosis [6]. The integration of focused physical examination techniques into complex clinical procedures such as ward rounds appears to be particularly marked by difficulties [7]. Even with respect to frequent clinical procedures such as IV cannulation, PY students show serious shortcomings, meaning that an independent implementation in the sense of an “entrustable professional activity” [8] is called into question even at the time when students are completing their medical education [9].

Physical examination techniques and clinical procedures are learned among the students themselves, with standardized patients, mannequins, part-task trainers, or directly at the patient’s bedside. With regard to the acquisition of physical examination competences, students prefer to learn with standardized patients or real patients; students only prefer learning with standardized patients for genital examinations [12]. Despite the continued widespread practice of the “see one – do one” approach [13], learning according to this approach appears to be ethically untenable for most clinical procedures due to their invasive nature. In this regard, in the framework of simulation-based medical education (SMBE) [14], simulation settings such as the “Skills Lab” [15] have established themselves and have proven to be effective for learning clinical procedures [5], [16], [17]. To enable this learning process to take place at a high level, continuous training using uniform faculty standards across all stages and areas of study is indispensable for the students. This is not least the case because a congruence has to exist between the assessment goals and criteria on the one hand, and the imparted learning goals and standards on the other, in the sense of a “constructive alignment” [18]. This should enable the learning incentives triggered by clinical assessments to be optimally utilized (“assessment drives learning”; [19]).

The central objective of the project “Heidelberg standard examination” was to establish uniform interdisciplinary standards for physical examination and clinical procedures in coordination with all clinical disciplines at the Heidelberg University Hospital. Further aims were to provide multimedia materials which would be available to assist all students from the first to the last day of their medical training, and to integrate the examination standards into the curriculum. At the same time, to foster the implementation into the curriculum, all lecturers working in the area of clinical teaching should have the same multimedia materials at their disposal. The standardization of medical examination techniques and the integration of the new joint standards into the curriculum should meet the needs of students as frequently expressed in student evaluations, sustainably increase the quality of student training and the acquisition of competences, and improve patient care. The following sections describe this process of interdisciplinary development of the teaching standards and the integration into the curriculum.

## Methods

### Project initiation and project team

The project arose in 2007 from a student initiative which the last author (MK) assisted and supervised with regard to content. Initially, a central objective of the project was to provide films on physical examination and clinical procedures created by students for students. These should serve as a helpful orientation in the preparation for clinical-technical examinations (objective structured clinical examination – OSCE; [20]) at the Heidelberg Medical Faculty. This student initiative led to plans to establish faculty-wide standards for these areas. As it developed further, the project was medically guided mainly by the University Hospital Department of Surgery (MK) and the University Medical Center (CN) and the project plans were implemented within a project and editorial team (co-authors).

#### Tasks of the project and editorial team

The composition and personal strengths of the project and editorial team varied over the running time of the project. Mostly, two to three medical personnel from the Departments of Surgery and Internal Medicine acted together to develop the project plans further. The tasks of the project and editorial team included coordinating team meetings, producing checklists, leading the interdisciplinary meetings with subject experts, developing layout suggestions, creating graphics and pictures, and implementing the texts in InDesign^©^. With regard to the filming of physical examination techniques and clinical-technical procedures, the project team was responsible for coordinating the film shoots, cutting and sound, and the final inspection by the experts. Advertising, clarification of legal aspects and funding applications should also not be forgotten.

#### Faculty-wide interdisciplinary coordination of learning goals and establishment of teaching standards

**Figure 1:**
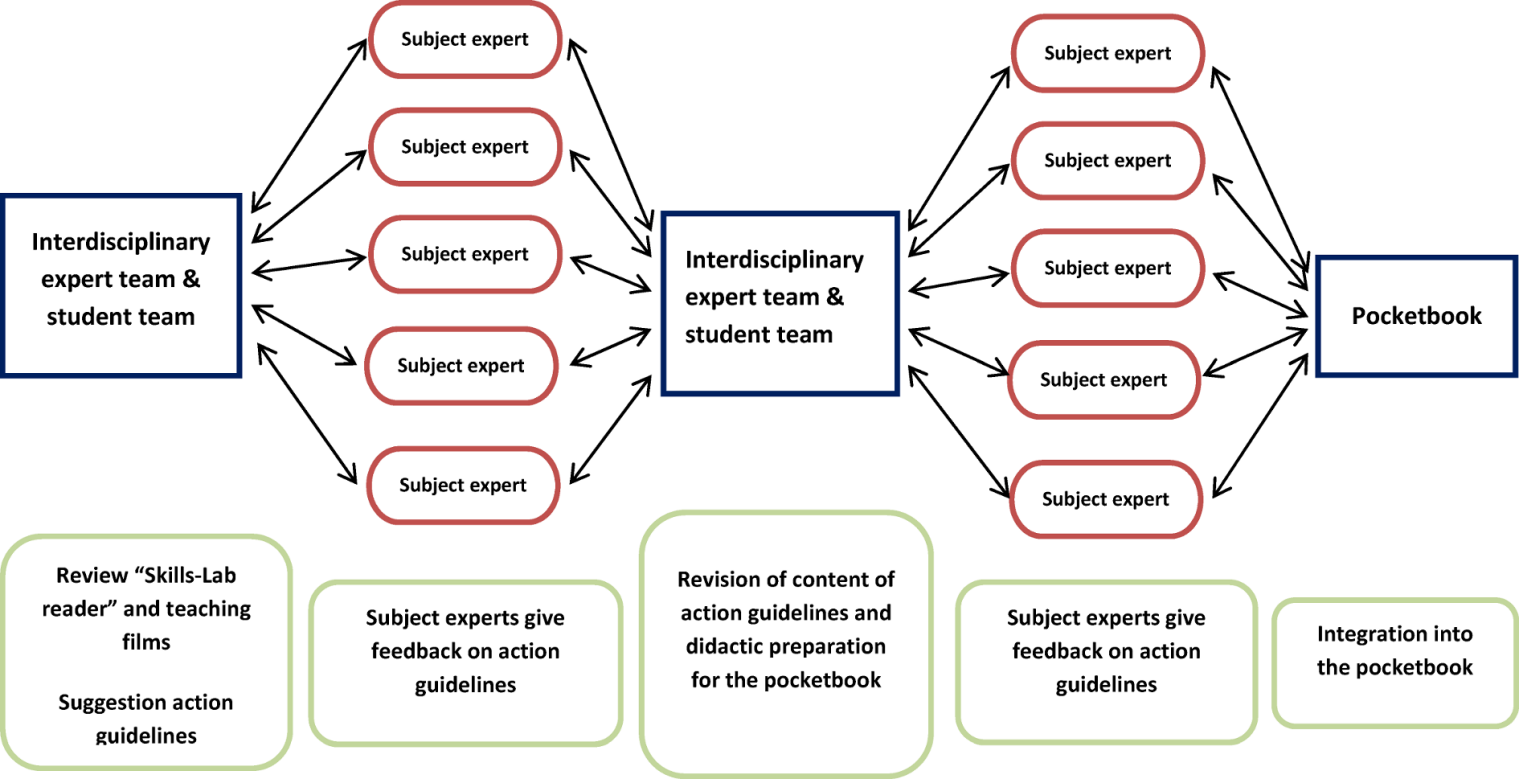
Iterative coordination process on learning goals and teaching standards (simplified schematic representation)

In an iterative coordination process between the subject areas of general medicine, ophthalmology, surgery, orthopedic medicine and trauma surgery, dermatology, geriatrics, gynecology, ENT medicine, emergency medicine, hygiene, internal medicine, neurology, neurosurgery, pediatrics, psychiatric and psychosomatic medicine, pathology, forensic medicine and urology, authorized medical representatives of the individual disciplines (“subject experts”) agreed on a uniform process for a standardized “head-to-toe” basic examination of entrusted patients, which was valid across all faculties. Moreover, for the individual physical examination techniques as well as clinical procedures which are used in several disciplines, all subject experts from these disciplines were included in the coordination process for agreeing upon learning goals and establishing teaching standards. For those physical examination measures or procedures which are only conducted in one of the disciplines, the respective subject expert developed, in coordination with his clinical department, the learning goals and the teaching standards to be established together with the project and editorial team. The iterative coordination process completed in each case is illustrated in figure 1 (Fig. 1).

#### Multimedia documentation and distribution of the faculty-wide standards

In consultation with the Dean of Studies of the Medical Faculty of the University of Heidelberg, the project and editorial team resolved and strived to distribute the developed teaching standards for physical examination techniques (“Heidelberg standard examination”) and for clinical procedures (“Heidelberg standard procedures”) in the form of a book and likewise to make them accessible in the form of film material. In this respect, each one of the 1,926 medical students of the Heidelberg Medical Faculty should be provided with these standards at no charge in the form of a pocketbook, and the films should equally be freely accessible on a homepage, together with the text and picture material in the book and the contents of the book chapters. At the same time, all medical lecturers involved in teaching should also receive a copy of the pocketbook.

#### Integration into the curriculum

The “Heidelberg standard examination” – represented by the developed pocketbook and the corresponding film sequences – was to be integrated into the curriculum from the preclinical part of studies up to the final examinations. To achieve this, a close consultation regarding the joint standards occurred with the course leaders of all existing examination courses: AaL-Plus (living anatomy) in the pre-clinical stage, interdisciplinary clerkship in surgery and internal medicine at the beginning of the clinical stage, clinical examination courses using standardized patients, bedside teaching (UaK) in all subject areas in the further clinical stage, and training in the clinical environment, self-directed learning and feedback conversations with the students during the practical year (PY). At the same, the clinical-technical examinations (OSCE; [21]), which had been revised and modified according to the faculty-wide standards, should serve the purpose of reviewing learning goals of the individual clinical subject areal.

#### Funding

Project funding was ensured by tuition fees and quality assurance funds of the Baden-Württemberg State Ministry for Sciences and Arts. From the beginning of the project in August 2009, two half-time physician positions as well as one student assistant position were continuously available, and from 2014 one half-time position for a film technician was provided. Moreover, the printing costs for the student copies of the pocketbooks “Heidelberg standard examination” and “Heidelberg standard procedures” (each approx. 35,000 €) as well as the costs for producing the homepage (approx. 8,000 €) were also covered. Sales of the pocketbooks by Heidelberg Medical Faculty also brought further funding opportunities for the project. The administration of the Heidelberg University Hospital pledged to fund the copies made available to the medical lecturers.

#### Gathering and integrating feedback

Feedback on the pocketbook “Heidelberg standard examination” can be provided through email addresses listed in the book. These user comments form the basis for a continuous revision and adaptation of the pocketbook.

## Results

### Pocketbook “Heidelberg standard examination”

**Figure 2:**
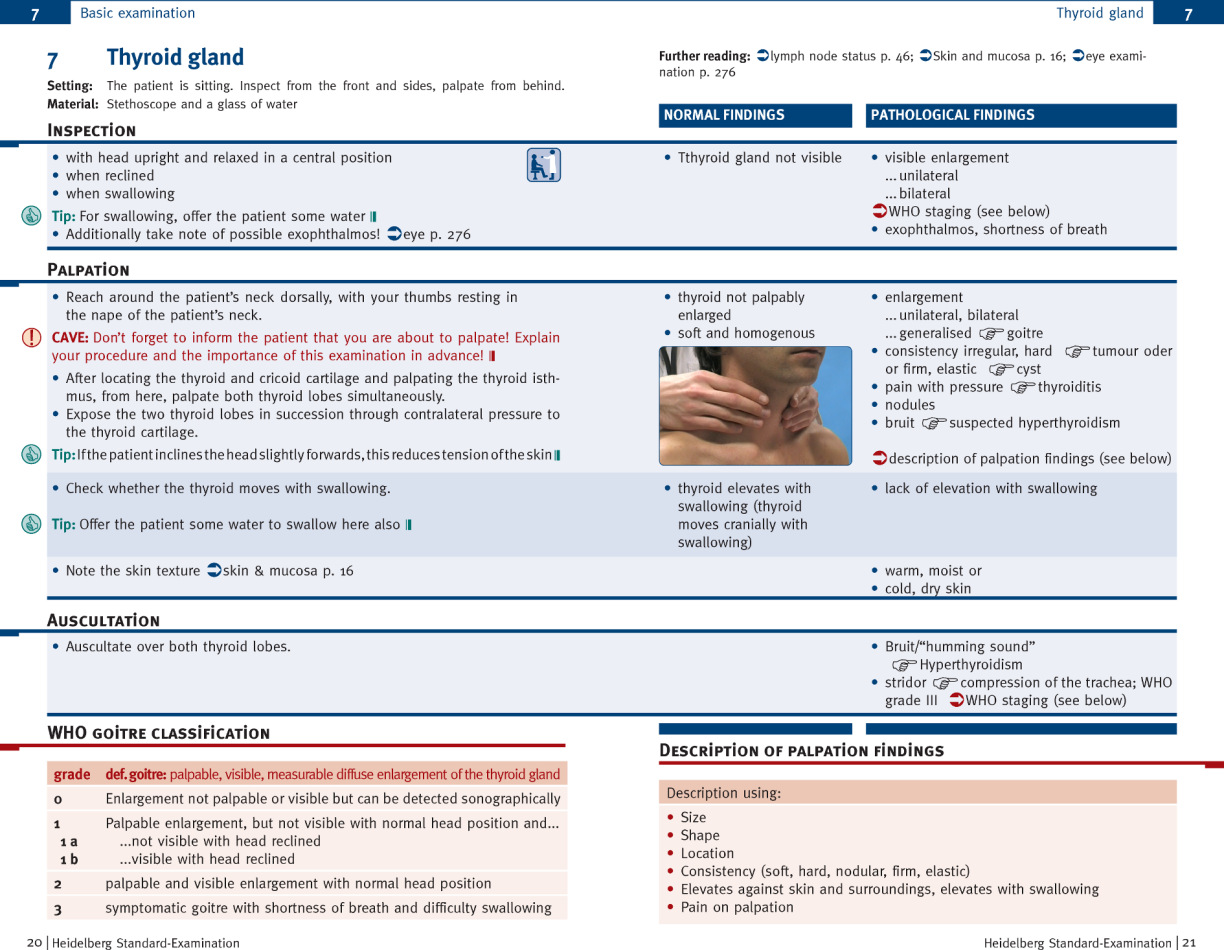
Example excerpt from the pocketbook “Heidelberg standard examination”

Figure 2 (Fig. 2) shows, by way of example, an excerpt from the pocketbook “Heidelberg standard examination” [22]. For presenting the physical examination techniques, a double-page format was chosen. Unique in its presentation, on the left-hand side there is a description of the relevant action steps, and on the right-hand side a comparison of possible normal and pathological findings. This provided the students, beyond the implementation of techniques, with support in using the correct nomenclature, description and documentation of normal and pathological findings. Additionally, “tips” were integrated, with advice on facilitating the practical procedure, warnings (“CAVE”) regarding complicating behavior, and boxes with clinically relevant information.

#### Pocketbook “Heidelberg standard procedures”

The graphical representation within the pocketbook “Heidelberg standard procedures” is illustrated in Attachment [23]. Besides a detailed description of the setting, the indications and contraindications for the respective clinical procedure, the required material, the relevant preparatory measures and the practical implementation are listed. The learner is also supported with “tips” and “CAVE” hints in the book.

#### Internal and external distribution

The first edition of the “Pocketbook Heidelberg standard examination” went to press in September 2012. This was distributed to all 1,926 students of the Medical Faculty, University of Heidelberg, as well as all 1,584 medical lecturers working in the faculty. The current financial basis allows all new students and medical lecturers to be equipped with a pocketbook in the subsequent three years. The external distribution is ensured through the homepage http://www.heidelbergerklinischestandards.de. We expressly chose not to cooperate with a textbook publisher in order to retain the rights for the contents and graphical material. In addition to sales of individual copies, 13 German and Austrian faculties were provided with large package orders. Besides the local provision of the Heidelberg students and lecturers, these external individual copy sales led to a turnover of approx. 500 books, and approx. 10,800 books were sold to other German-speaking faculties. The “Heidelberg standard procedures” pocketbook went to press in December 2015 [23].

#### Film hosting

**Figure 3:**
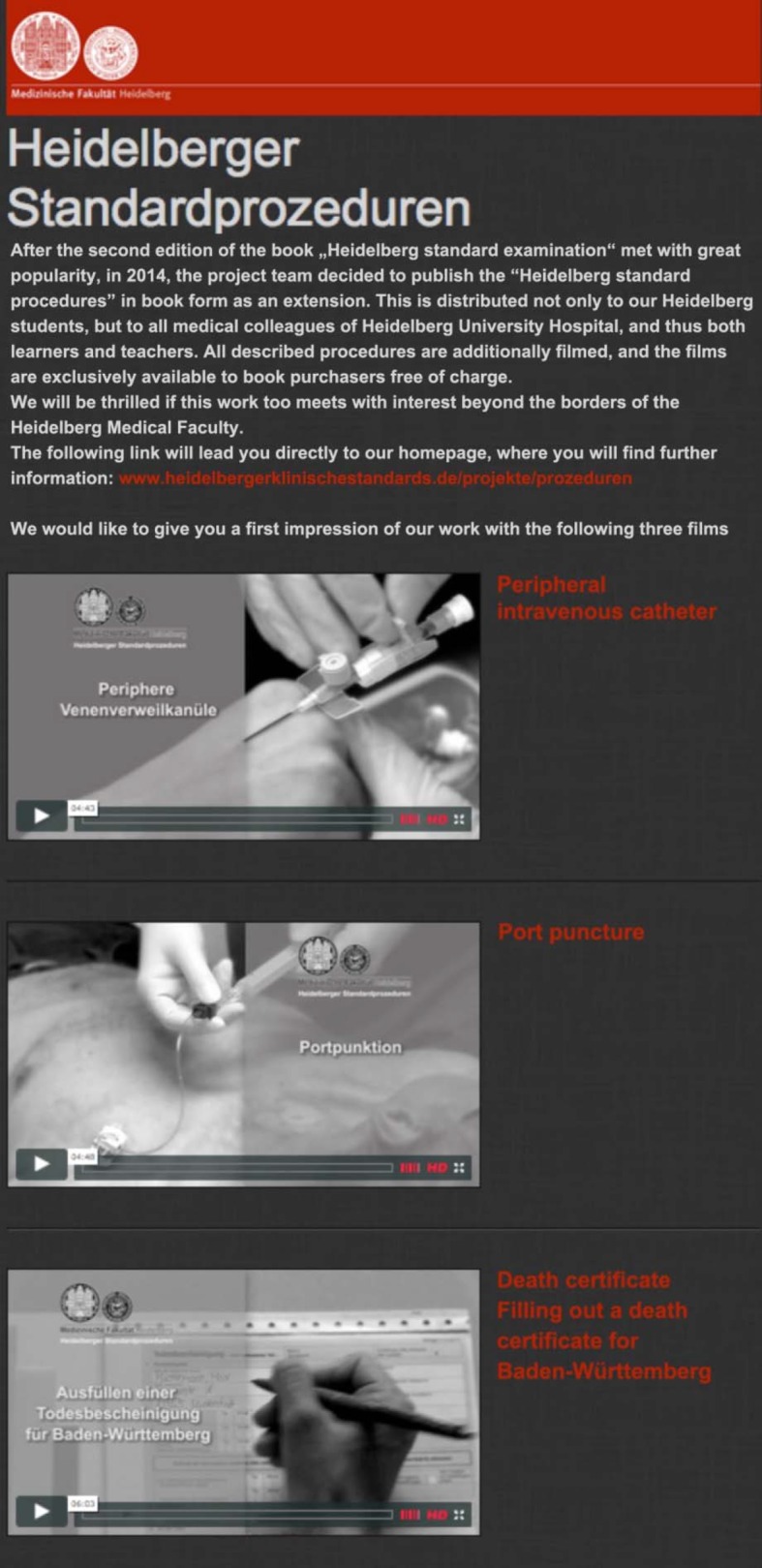
Sample sequences of the film material for “Heidelberg standard procedures” presented on http://www.heidelbergerklinischestandards.de/projekte-video/prozeduren/

The films corresponding to the physical examination techniques and clinical procedures are presented on the homepages http://www.heidelbergerklinischestandards.de. All purchasers of the pocketbooks “Heidelberg standard examination” and “Heidelberg standard procedures” can freely access the film material. Sample sequences can be found at http://www.heidelbergerklinischestandards.de/projekte-video/untersuchungen/ and http://www.heidelbergerklinischestandards.de/projekte-video/prozeduren (see figure 3 (Fig. 3)).

#### Integration into the curriculum

Table 1 (Tab. 1) shows the time points in the curriculum of the University of Heidelberg Medical Faculty at which a student receives learning units on physical examination. Furthermore, the time points of the relevant clinical-technical examinations are given.

#### Feedback

Through the correspondence address provided in the pocketbook, abundant constructive criticism was given on the part both of the students and the lecturers of the Medical Faculty of Heidelberg University. These were consistently positive and referred to the didactic design (“[…]. The presentation…of this book is great”, You’ve created a super layout”), the quality of the content (“[…]. A brilliant work! Until now I had laboriously compiled the info on examinations myself. Now it’s been done for me...”) and the film realization (“In combination with the […] teaching videos it’s simply great!”) as well as points of criticism with regard to medical content.

## Discussion

In the current project description, we presented the process of developing faculty-wide standards for clinical physical examination and clinical procedures at the University of Heidelberg Medical Faculty. We presented a description of the objectives, the logistical coordination of learning goals and development of the teaching standards, as well as the integration into the curriculum and distribution in the form of the pocketbooks “Heidelberg standard examination” [22] and “Heidelberg standard procedures” [23] and of the online film material, which is automatically accessible upon acquisition of the pocketbooks.

Based on the local resonance and the re-evaluation of the project, the initiative can be deemed a success. The feedback which the project team received through the email addresses provided in the pocketbook is consistently positive. Constructive points of criticism regarding content and suggestions for improvement were gratefully accepted and incorporated in the 2nd edition of the pocketbook “Heidelberg standard examination”. Substantial orders from German and international German-speaking medical faculties, and strong demand for the pocketbook “Heidelberg standard examination” from private individuals also illustrate that the pocketbook and the corresponding film material close a gap in demand. A 2nd, improved edition of the pocket book “Heidelberg standard examination” has since been printed. Currently, the corresponding films are being produced and completed in the accompanying online offer. The pocketbook “Heidelberg standard procedures” went to press in December 2015. The film production for the presented procedures has been completed for this subject area and will be fully available when the book is distributed, and in turn made available to all students and medical lecturers of the University of Heidelberg Medical Faculty. Sample videos can be viewed at http://www.heidelbergerklinischestandards.de/projekte-video/prozeduren/. Further development of contents is planned in the coming years for the areas of complex clinical procedures (e.g. anesthesia induction, conducting ward rounds etc.).

Besides the distribution of the multimedia implementation of the teaching standards for physical examination and clinical procedures, the integration into the curriculum and into assessment constitutes a challenge. This is implemented in the form of a longitudinal networking of learning contents on physical examination with clinical procedures, which spans from the first semester to the PY. The continuous needs assessment, adjustment of learning goals, evaluation and curriculum adaptation and quality assurance should certainly be seen in this regard as a long-term process in the sense of a Kern cycle [1].

### Limitations

In terms of limitations, it should be noted that the introduced university standards are based upon expert consensus as an evidence base is currently lacking. Moreover, although the feedback on the project is very positive, it was only requested unsystematically via email within the pocketbook. However, the focus and a potential strength of the current project report can be found in the detailed description of the creation process. This could enable other faculties with a similar intention to gather stimuli and orientation. A particular strength of the project itself is, in our view, the central incorporation of highly motivated students, who helped shape the project to a very high degree, and who were rewarded for this with the GMA prize for teaching students in 2010.

## Conclusions

Overall, the whole process of developing uniform teaching standards for physical examination and clinical procedures has proven to be laborious but worthwhile. It has provoked an important curriculum-based stimulus, an interdisciplinary discourse regarding subject and content, and provides the students with greater security in terms of behavior and examination. 

## Competing interests

The authors declare that they have no competing interests.

## Supplementary Material

Example excerpt from the pocketbook “Heidelberg standard procedures”

## Figures and Tables

**Table 1:**
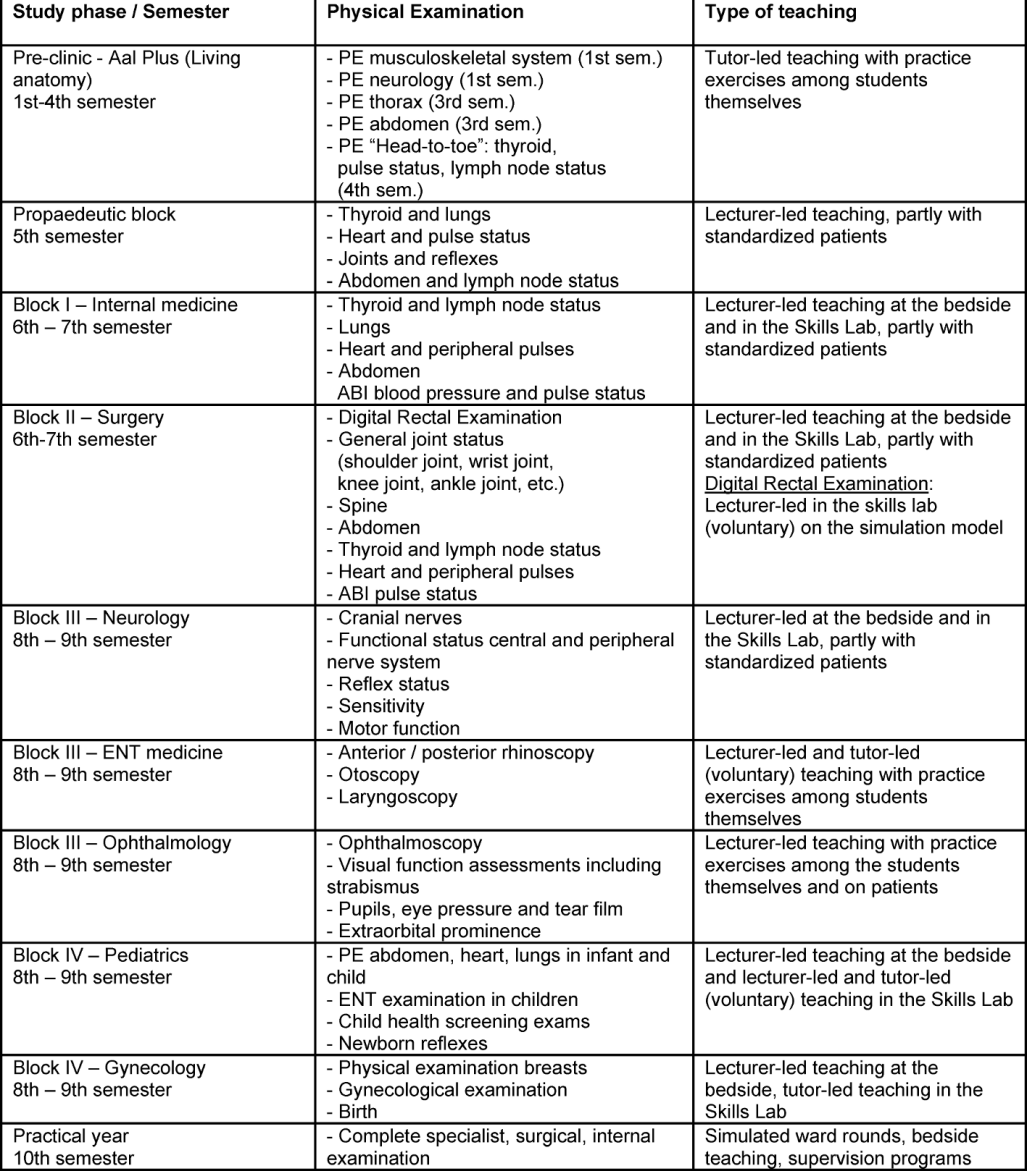
Integration into curriculum using the example of teaching of competences for physical examination
