# Supplementary material for: “Heidelberg standard examination” and “Heidelberg standard procedures” – Development of faculty-wide standards for physical examination techniques and clinical procedures in undergraduate medical education
Source: GMS J Med Educ. 2016 Aug 15;33(4):Doc54. doi: 10.3205/zma001053 (PMC5003136; doi:10.3205/zma001053)

23.1 Surgical hand disinfection

Setting

Surgical hand disinfection is mandatory before all surgical procedures. This applies to all surgical fields. Its aim is to **eliminate transient skin flora** and to **reduce residual skin flora** as much as possible. A washroom is available in the operating area for surgical hand disinfection.

CAVE: It is forbidden to wear jewellery and polished/artificial finger nails. Wearing artificial finger nails in particular has proven to be a source of nosocomial infections. There must be no marked injury to the nail bed or inflammations.

Note: The finger nails of all persons acting in operations should be kept short and round.

Indications

- Every surgical procedure
- before wearing sterile gloves (mikropores! ➲ S. 20)

Material

① Liquid soap in the dispenser
② Hand disinfectant in the dispenser
③ Sink
④ Low-germ disposable hand towels
⑤ Clock*

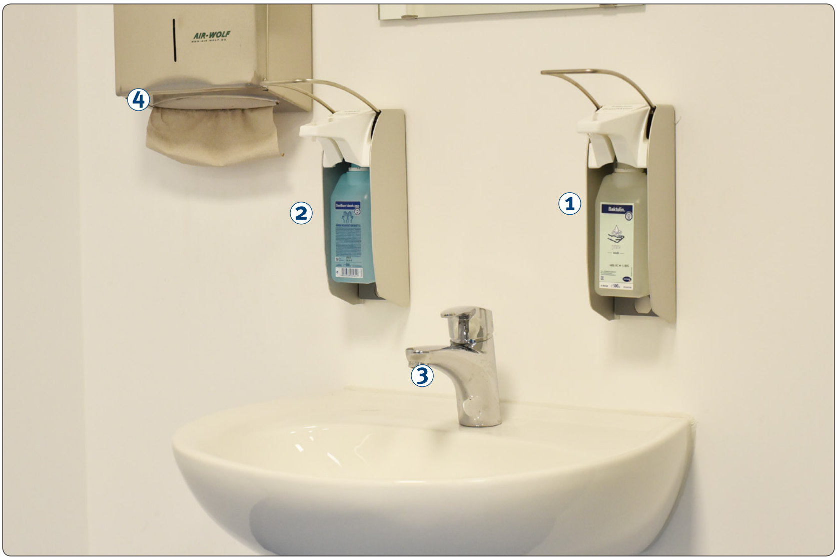

Potential complications

- Skin irritations
- Allergic reactions

Preparation

Soap and disinfectant

- Ensure that there is sufficient and suitable soap and disinfectant in the dispenser.

Implementation of surgical hand disinfection

Hand washing

- Apply a sufficient amount of soap.
- Wash hands and forearms **up to the elbows for at least one minute** (1).
- Point the fingertips upwards and the elbows lower, enabling the water to flow down to the elbows. Make sure that you wash all areas, **in particular the spaces between the fingers** (2).
- If necessary, nails and nail folds should be brushed with a soft, sterile synthetic brush and soap. Brushing skin and forearms leads to increased skin irritations and increased release of germs! **A brush should only be used in the case of extensive soiling!**
- Then wash off the soap so that the water flows down to the elbows (3).
- Dry off the hands and forearms with a low-germ disposable hand towel.

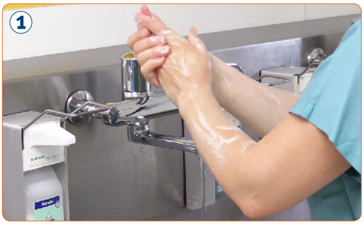

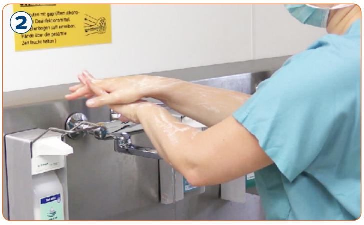

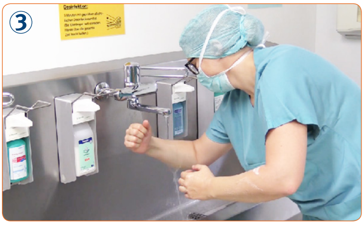

Note: Hand washing is necessary prior to carrying out the first surgical hand disinfection of the day. If several operations take place in quick succession (break between operations <60 minutes), with a low likelihood of contamination (intact gloves), re-washing the hands is not necessary and surgical hand disinfection can ensue directly.

Hand disinfection

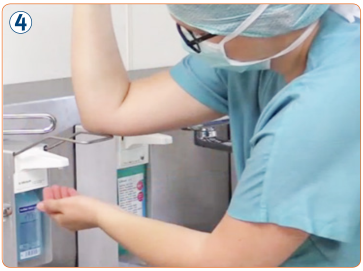

- Do not use the disinfectant dispenser with the hands. It can be used easily with the **back of the elbows** (4).
- Apply a **generous amount** of disinfectant to one hand.
- Carry out disinfection for at least **90 seconds**.

Implementation of surgical hand disinfection (continued)

Hand disinfection (continued)

- Distribute the disinfectant across both hands and forearms up to the elbows (⑤).
- Take care to rub the disinfectant thoroughly into all areas and keep them moistened for the whole duration.
- Make the same movements as done for the **hygienic hand disinfection** ➲ S. 18. Pay particular attention to the ...
  - **thumbs** (⑥)
  - **spaces between the fingers** (⑦)
  - **sides of the hands**
  - **edges of the hands**
- During the **90 seconds**, repeatedly apply fresh disinfectant to the hands if you notice that they are becoming dry.
- Continue to keep the hands and forearms in the above-mentioned position:
  - with the hands and fingertips pointing upwards,
  - the hands at approx. chest height,
  - and the elbows downwards (⑧).
- After **90 seconds**, allow the hands and forearms to dry, and go into the operating room.
- It is not allowed to dry them off with a towel again.
- The hands and forearms should be dry before you put on the surgical scrubs and sterile gloves, as otherwise skin irritations can occur more easily and hygiene is limited (wet sleeves!).

⑤
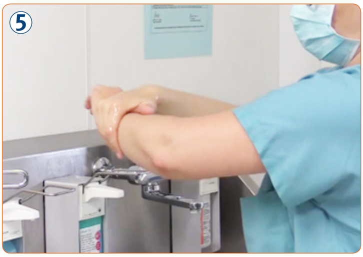

⑥
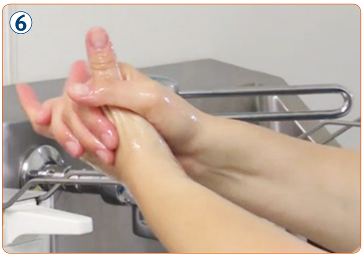

⑦
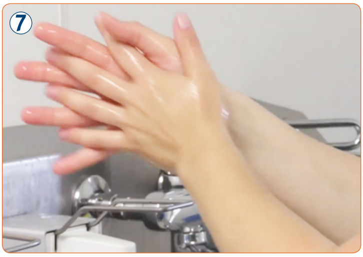

❗ **CAVE:** Make completely sure that you do **not touch any unsterile surfaces or objects**. If this happens, you will have to **begin disinfection again**.

⑧
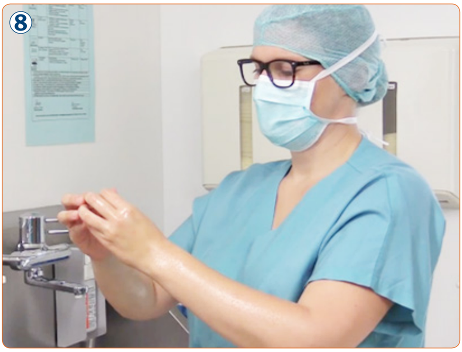

Supplement: Example excerpt from the pocketbook “Heidelberg standard procedures” [file JME-33-54-s-001.pdf]
